# Prokaryotic taxonomy and nomenclature in the age of big sequence data

**DOI:** 10.1038/s41396-021-00941-x

**Published:** 2021-04-06

**Authors:** Philip Hugenholtz, Maria Chuvochina, Aharon Oren, Donovan H. Parks, Rochelle M. Soo

**Affiliations:** 1 Australian Centre for Ecogenomics, School of Chemistry and Molecular Biosciences, The University of Queensland, Brisbane, QLD Australia; 2 Department of Plant and Environmental Sciences, The Alexander Silberman Institute of Life Sciences, The Edmond J. Safra campus, The Hebrew University of Jerusalem, Jerusalem, Israel

**Keywords:** Archaea, Bacteria

## Abstract

The classification of life forms into a hierarchical system (taxonomy) and the application of names to this hierarchy (nomenclature) is at a turning point in microbiology. The unprecedented availability of genome sequences means that a taxonomy can be built upon a comprehensive evolutionary framework, a longstanding goal of taxonomists. However, there is resistance to adopting a single framework to preserve taxonomic freedom, and ever increasing numbers of genomes derived from uncultured prokaryotes threaten to overwhelm current nomenclatural practices, which are based on characterised isolates. The challenge ahead then is to reach a consensus on the taxonomic framework and to adapt and scale the existing nomenclatural code, or create a new code, to systematically incorporate uncultured taxa into the chosen framework.

## Introduction

Naming and classifying the world around us is a natural human prerogative for effective communication [1]. With regard to the biological sciences, formal structures first arose in the 1700s through the work of Linnaeus [2]. Linnaeus introduced the principles of modern biological taxonomy (arrangement of plants and animals into hierarchical categories) and nomenclature (rules for naming taxonomic groups of plants and animals), which today form the basis of biological classification. Originally taxonomy was based on shared properties (chiefly anatomical, but also biochemical and physiological), developmental processes (e.g., live birth vs. eggs) and behaviours (e.g., flight), later collectively termed phenotype to distinguish these features from hereditary information (genotype) [3]. This intuitively reflected the concept of common ancestry even though evolutionary theory had yet to be developed at the time of Linnaeus. This works quite well for animals and plants with a few celebrated red herrings, such as the long-held belief that hippos were most closely related to pigs based on anatomical similarities; genotype indicates that they are actually more closely related to whales [4]. Phenotype was also used for decades to classify microorganisms despite much less conspicuous morphological and developmental traits than animals and plants [5]. However, phenotype provides little insight into deep evolutionary relationships of microorganisms, which can only be discerned by comparison of conserved information-bearing macromolecules [6]. Moreover, the realisation that most microbial diversity had been overlooked because most microbes cannot easily be grown in the laboratory has further hamstrung microbial classification [7–9]. This review concerns microbial taxonomy and nomenclature with a primary focus on Bacteria and Archaea, from an historical perspective to modern day, and an exploration of how recent advances in culture-independent genome sequencing may be harnessed to provide a comprehensive and systematic classification of the microbial world.

## Taxonomy: improving the framework

Taxonomy is most commonly defined in biology as the branch of science, which names and classifies organisms based on shared properties [10, 11]. However, here we define taxonomy according to its original Ancient Greek derivation as *táxis* for ‘order or arrangement’ and *nomos* meaning ‘law’ typically manifested as a hierarchical structure or framework in biology. We specifically exclude nomenclature from this definition, i.e., formal naming schemes and rules, which govern them, discussed separately below. We do this because taxonomy (thus defined) and nomenclature can and have operated independently, particularly in microbial classification, which can create conflicts (see below).

Taxonomy can be based on any combination of properties; however, beginning with Darwin’s recognition of common descent, biologists now agree that taxonomy should be based on evolutionary relationships as the most natural way of arranging organisms [12]. In this regard microorganisms have until recently been the most problematic taxa to arrange in a phylogenetic framework because their phenotypic properties for the most part do not reveal their common ancestry [6].

### Phenotypic classification

The first modern attempt to systematically classify bacteria based on their phenotypic properties began with the first edition of Bergey’s Manual of Determinative Bacteriology in 1923, which categorised bacteria into a nested hierarchical classification to indicate differing levels of relatedness. Initially this comprised from highest (most distantly related) to lowest (most closely related) rank; class, orders, families, tribes, genera and species based on identification keys and tables of distinguishing characteristics [13]. The keys relied heavily on morphology, culturing conditions and pathogenic characters with the primary goal being practical identification of isolates at the species level rather than constructing an evolutionary framework. Numerical taxonomy, proposed by Sokal and Sneath in 1962 [14–16], provided a mathematical basis for quantitative comparisons of phenotypic properties between bacteria typically incorporating dozens of features. Although in principle, numerical taxonomy could incorporate phylogenetic information, in practice it was used primarily for identification and lacked a rigorous evolutionary framework. A heartfelt acknowledgement of the limited evolutionary resolution afforded by phenotypic characteristics was made on numerous occasions by Stanier and van Niel in the 1940s–1960s [17–19], where they concluded that it was a waste of time for taxonomists to attempt a natural system of classification (i.e., one based on evolution) for bacteria. However, it was during this period that the path forward to breaking the phenotype impasse was predicted by Zuckerkandl and Pauling through the use of informational macromolecules that could act as molecular clocks to infer evolutionary relationships [20].

### Small subunit ribosomal RNA, the molecular pioneer of microbial classification

Inspired by the work of Zuckerkandl and Pauling, Woese began a search for a molecular chronometer that could form the basis of an evolutionary framework for all life. He landed upon the ribosome as a good candidate, most famously the small subunit ribosomal RNA (16S/18S rRNA) contained therein, due to its high sequence conservation holding together the structural core of the ribosome, interspersed with variable regions not under the same exacting selective pressure. The combination of these properties make small subunit rRNAs useful molecular clocks with both an hour and minute hand to measure ancient and more recent relationships [21–24]. Several other DNA-based classification methods have been developed over time, including DNA–DNA hybridization [25, 26], DNA G + C content [27, 28], pulsed-field gel electrophoresis [29, 30], and more recently multilocus sequence typing [31, 32] and multilocus sequence analysis [33, 34]. However, like their phenotypic predecessors these methods are not useful for deep phylogenetic reconstructions, whereas comparative analysis of small subunit rRNAs is able to provide an objective evolutionary framework across the tree of life. The highlight of Woese and his colleagues’ analyses was the discovery of Archaea [21] completely overlooked by identification keys because of the inability to frame phenotypic properties such as methanogenesis in the correct phylogenetic context [35].

The 16S rRNA gene was also instrumental in highlighting the enormous amount of microbial diversity missed by culturing methods [7, 11, 36]. Pace and colleagues were the first to characterise microorganisms via their 16S rRNA sequences obtained directly from the environment through the ingenious use of highly conserved ‘universal’ primers broadly targeting this molecule [22]. These primers were subsequently used to PCR-amplify 16S rRNA genes from extracted genomic environmental DNA. Mixed amplicons were then cloned and sequenced to provide profiles of the in situ microbial community [37]. As sequencing technologies improved, the cloning step could be omitted, and thousands of samples from dozens of habitats were readily profiled [38–40], which brought with it a plethora of databases and tools for analysing and classifying 16S rRNA gene sequences (Table 1). By the end of the 1990s, the redefining of prokaryotic taxonomy through the lens of 16S rRNA sequences was sufficient to induce Bergey’s Manual Trust to transition from traditional phenotype-based classification to a 16S rRNA-based phylogenetic framework in the second edition (2001–2012) of Bergey’s Manual of Systematic Bacteriology [41].

**Table 1:** Online taxonomic and nomenclatural resources.

Name of resource	Tax/Nom^a^	Type (16S, G,M)^b^	Taxonomic scope	Number of sequences in current release	Year commenced	Hyperlink to resource	References
RDP	Tax	16S/28S	Bacteria, Archaea, Fungi	RDP Release 11 3,356,809 16S 125,525 28S	1992	https://rdp.cme.msu.edu/	[123, 124]
SILVA	Tax	16S/18S, 23S/28S	Bacteria, Archaea, Eukaryotes	Silva SSU/LSU 132 6,073,181 SSU 907,382 LSU	2008	https://www.arb-silva.de/	[125–127]
EzBioCloud	Tax	16S, G	Bacteria, Archaea	Aug 06, 2019 81,189 taxa 64,416 16S 146,704 genomes	2010	https://www.ezbiocloud.net/	[128]
Greengenes	Tax	16S	Bacteria, Archaea	Out of commission	2006–2013	https://greengenes.secondgenome.com/	[129, 130]
MIDAS	Tax	16S	Bacteria, Archaea	Jun-2020 4,245 species	2015	http://www.midasfieldguide.org/	[131, 132]
NCBI	Tax	16S,G	Bacteria, Archaea, Eukaryotes, Metazoa, Viridiplantae, Viruses	Jun-2020 905,918 species	1993	https://www.ncbi.nlm.nih.gov/taxonomy	[133]
GTDB	Tax	G	Bacteria, Archaea	05-RS95 194,600 genomes	2018	https://gtdb.ecogenomic.org/	[44]
TYGS	Tax	G	Bacteria, Archaea	Jun-2020 11,819 genomes	2019	https://tygs.dsmz.de/	[134]
JGI IMG	Tax	G	Bacteria, Archaea, Eukaryotes, Virus	Aug-2019 104,759 genomes	2006	https://genome.jgi.doe.gov/portal/	[135, 136]
IJSEM	Nom	16S,G,M	Bacteria, Archaea		1951	https://www.microbiologyresearch.org/content/journal/ijsem	[5]
LPSN/DSMZ	Nom	16S,G	Bacteria, Archaea	May-2020 18,678 16S, 77,990 strain deposits	1997	https://lpsn.dsmz.de/	[112, 137, 138]
Namesforlife	Tax, Nom	16S,G	Bacteria, Archaea	Sep-2019 16,335 16S, 10,877 genomes	2004	https://www.namesforlife.com/	[139]
Cyanotype	Tax	16S	Cyanobacteria	386 strains	2017	http://lege.ciimar.up.pt/cyanotype/	[140]
CyanoDB	Tax,Nom	16S,G,M	Cyanobacteria	Sep-2019 1635 taxa	2004	http://www.cyanodb.cz/	[141]
AlgaeBase	Tax,Nom	16S/18S,G,M	Algae, Cyanobacteria	Sep-2019 156,143 species	1996	https://www.algaebase.org/	[142]
StrainInfo	Tax, Nom	16S,G,M	Bacteria, Archaea, Fungi	Out of commission	2014–2018	http://www.straininfobreak.ugent.be/	[143]

^a^Tax (Taxonomy), Nom (Nomenclature).

^b^16S/18S/23S/28S (16S/18S/23S/28S rRNA gene), G (Genome), M (Morphology).

Polyphasic taxonomy emerged as an approach to integrate phenotypic and genotypic characteristics in order to produce a consensus taxonomy that best reflected the many and varied attributes of biological organisms [10]. The original definition of polyphasic taxonomy by Colwell in 1970 predated and made no reference to phylogenetic inference, but with the advent of 16S rRNA analysis, phylogenetic classification rose to prominence [42]. Due to the high sequence conservation of the 16S rRNA gene, polyphasic taxonomy was stratified such that 16S rRNA trees informed classifications at and above the rank of genus, whereas species and subspecies level delineations were better accommodated by chemotaxonomic methods such as multilocus enzyme electrophoresis and whole-cell protein analysis, and more recently by comparison of genome sequences [26, 42, 43]. The advent of whole-genome sequencing, and its rapid acceleration in recent years due to technological advances has provided increasing impetus for bacterial and archaeal taxonomy to transition again, this time from a 16S rRNA-based to a genome-based classification [44, 45].

### Genome-based classification

As with the 16S rRNA gene, genome sequences can be used to construct a robust phylogenetic framework on which to base a systematic classification [44]. Enormous advances in both high-throughput sequencing and high-performance computing have enabled sequenced genomes to form the basis of a classification framework. Genome-based classification affords greater resolution than the 16S rRNA gene (which represents only 0.05% of an average 3-Mbp prokaryotic genome) for both the most ancient and most recent relationships due to a larger fraction of the genome being used in the comparison, which provides an improved phylogenetic signal [46–48]. However, since most gene families have some history of horizontal gene transfer between organisms, genome-based phylogenies typically use a subset of conserved vertically inherited genes as the basis of the inference [49–51]. A notable exception is the rank of species for which methods using much greater fractions of the genome have been developed (Box 1). Two main approaches exist for building evolutionary trees from genome sequences; supertrees and supermatrices. In the construction of supertrees, independent gene trees are created and then combined to produce a single, consensus estimate of phylogenetic relationships between organisms [52–54]. Supermatrices involve concatenating genes into a single phylogenetic matrix of aligned sequences from which the tree is then inferred [47, 55–57]. Both methods have been used successfully to infer phylogenies across the tree of life, and in a recent direct comparison of a bacterial supertree and supermatrix, had a 98.2% taxonomic congruence despite being based on different sets of marker genes [58]. Other classification methods, which make use of genome sequences include similarity measures between pairs of genomes either at the level of encoded proteins (average amino acid identity) [59], or nucleotides (average nucleotide identity (ANI)) [59, 60] and digital DNA–DNA hybridisation [61, 62]. However, these methods do not use an explicit evolutionary model like supertrees and supermatrices and are used primarily for defining and identifying species (Box 1).

Like 16S rRNA sequences, genome sequences have been extended into the uncultured domain via shotgun sequencing of environmental samples. This metagenomic approach has also benefitted greatly from improvements in sequencing and computation, and today it is possible to recover near-complete or even complete genome sequences of naturally occurring microbial populations from environmental DNA, so-called metagenome-assembled genomes (MAGs) [63–65]. Indeed, the number of available MAGs is rapidly eclipsing the number of isolate genomes due to the relative ease of obtaining multiple MAGs from a single metagenome [9]. In instances where retrieval of genome sequences of low abundance or heterogeneous populations from environmental samples is not feasible, single cell genomics has advanced to the point where single-amplified genomes (SAGs) can represent such taxa [8, 66, 67]. This rapid accumulation of genome data from uncultured taxa raises an enormous challenge for classification, both in terms of taxonomic placement and nomenclature (see ‘Nomenclature: controlling the vocabulary’). It is estimated that uncultured taxa represent upwards of 85% of microbial diversity according to Faith’s phylogenetic diversity metric [8] meaning that taxonomic frameworks established over previous decades have major gaps in them. This issue is even more pronounced in the viral world with a recent estimate of 10^31^ bacteriophage in the environment represented by only a few thousand sequenced genomes [68].

It is widely recognised that prokaryotic taxonomy is riddled with phylogenetic inconsistencies (polyphyletic taxa) due to historical use of phenotypic data [69], chimeric 16S rRNA gene sequences from PCR-based environmental surveys [70], and premature conclusions based on phylogenetic reconstructions lacking suitable outgroups [71]. These problems have been compounded by the tidal wave of gene and genome sequences from uncultured taxa. Consequently, several databases and tools have been developed to try to address these shortcomings through the establishment of robust phylogenetic frameworks for microbial classification, firstly using 16S rRNA gene sequences, and more recently using genome sequences (Table 1). All of these resources face the same technical challenge of having to compare hundreds of thousands of sequences to each other to provide a global view of microbial diversity, which is difficult for individual genes and more so for genomes. However, common features of successful resources include computationally cheap dereplication of sequences and inference of a robust and scalable evolutionary framework. Whether these resources continue to scale with the rapidly increasing sequence database remains to be seen.

Historically, definition of ranks based on phenotypic data has been highly subjective, particularly for ranks above species. The introduction of gene and genome-based classification has provided the opportunity to define genus and higher ranks based on objectively quantifiable sequence similarities. In 2014, Yarza and colleagues proposed standardised thresholds for defining prokaryotic lineages from genus to phylum based on 16S rRNA gene sequence identities [11]. While certainly removing many inconsistencies in existing taxonomic classifications, and having the benefit of accommodating uncultured taxa, this approach does not take into account phylogenetic relationships and variable rates of evolution between lineages. As such, fast-evolving groups with more divergent 16S rRNA sequences are classified in higher than expected ranks, such as mycoplasma bacteria which constitute two phyla by this identity-based criterion. Vertebrate-associated mycoplasmas, however, are estimated to have diverged from their arthropod-associated sister lineage (ureaplasmas) only 400 Mya, which is much later than the estimated primary diversification of bacterial phyla (2–3 Gya) [44]. This issue can be offset by use of relative evolutionary divergence (RED) distances, which normalise for variable substitution rates across a phylogenetic tree [44]. After RED correction on a concatenated conserved marker gene tree, mycoplasmas were classified into a single order within the phylum Firmicutes more consistent with their estimated time of divergence from ureaplasmas, suggesting that this approach may be better suited for systematically defining higher ranks than uncorrected identity thresholds [44].

Finally, it is important to note that there is no official prokaryotic taxonomy to ensure freedom of taxonomic opinion, but also because underlying technologies used to define taxonomic hierarchies have been changing so rapidly [1, 72]. However, different taxonomies incorporating named prokaryotic isolates have been effectively linked through an official nomenclature.

Box 1 Species—a foundational taxonomic unit and biological entity Species are the cornerstone of both taxonomy and nomenclature; however, what constitutes a prokaryotic species has been widely debated over the years [3, 144–147]. For classification purposes, species definitions based on phenotypic properties have been necessarily practical using a combination of traits that together are deemed to be diagnostic of a species, but individually are often not unique to a given species such as cell morphology and use of different carbon sources. Since the discovery of DNA, more objective operational definitions of a species based on sequence similarity thresholds have been favoured, beginning with DNA:DNA hybridization of ≥70% [148, 149], 16S rRNA similarities of ≥97% [150] and most recently ANI of ≥95% [59, 60, 151, 152]. By contrast, a biological species concept has been widely applied in zoological taxonomy based on the ability of species to recombine their DNA (i.e., reproduce) with members of their own species, but not with members of other species [153]. It was recently proposed that this biological species concept could be extended to all lifeforms including asexually reproducing prokaryotes using their genome sequences [154]. By informatically identifying groups of bacterial strains that freely exchange genes through homologous recombination from those that do not, species were able to be circumscribed based on recombination barriers that did not necessarily conform to a fixed sequence similarity threshold [154]. Ultimately, taxonomies based on bona fide biologically defined species would be the best natural classification system. This would also be a step in the right direction for microbial ecologists who wish to address species as meaningful biological rather than operational units [146, 147].

## Nomenclature: controlling the vocabulary

### The development of nomenclatural codes

**Fig. 1:**
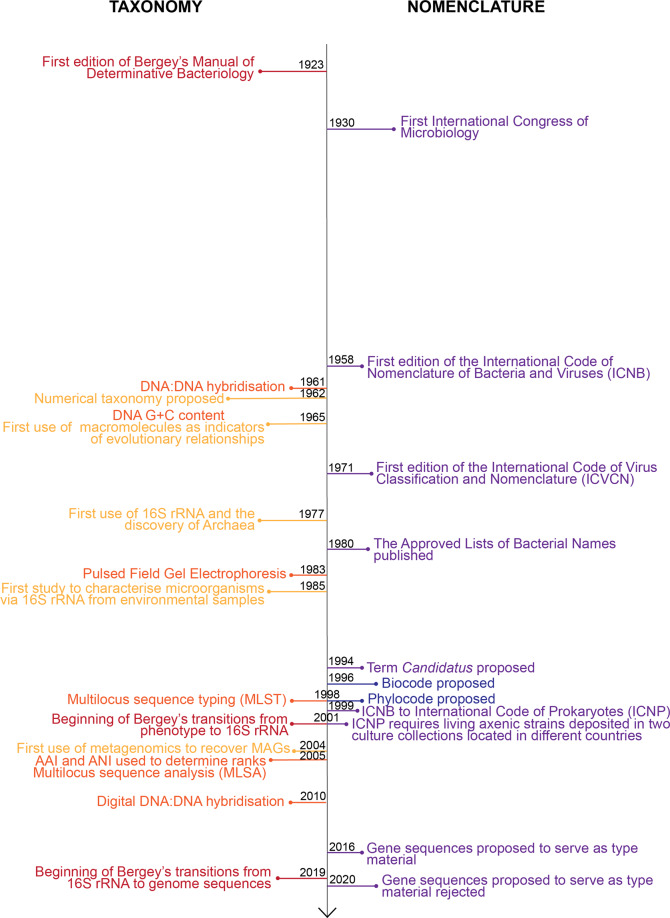
Key events in prokaryotic taxonomy and nomenclature over the past 100 years. Taxonomic events are shown in the left panel and nomenclatural events in the right panel. Time is shown on the vertical axis from 1920 (top) to present (bottom).

Nomenclature, the business of systematically naming things, was first proposed for biological entities (plants and subsequently animals) by Linnaeus in the mid 1700s in which he introduced the concept of a taxonomic hierarchy (described above). Most famously this included the binomial nomenclature system comprising the two lowest canonical ranks: genus and species [73]. His work became the foundation for hierarchical taxonomy in both botany and zoology with the establishment of nomenclatural codes over 100 years later, most recently called the International Code of Nomenclature for algae, fungi and plants (ICN or Botanical Code) founded in 1867 and International Code of Zoological Nomenclature (Zoological Code) founded in 1905, in which a set of rules for naming plants (and algae and fungi) and animals was laid out and controlled by elected committees of experts. Until 1947, microorganisms had been predominantly classified under the Botanical Code because bacteria had traditionally been considered fungi [74, 75]. In 1930 at the First International Congress of Microbiology in Paris, it was proposed that bacteria and viruses should have their own code, resulting in the Revised Edition of the International Code of Nomenclature of Bacteria and Viruses in 1958, today called the International Code of Nomenclature of Prokaryotes (Prokaryotic Code) [76] to reflect the inclusion of archaea and removal of viruses [77] (Fig. 1). One notable exception is the bacterial phylum Cyanobacteria, which is still mostly classified under the Botanical Code due to the association of oxygenic photosynthesis with plants (Box 2). Additional codes have been proposed for specific subsets of taxa including cultivated plants (ICNCP; 1952), viruses (ICVCN or Virus Code; 1966) and plant associations (ICPN; 1976) resulting in the six International Codes recognised today each controlled by a committee of experts (Fig. 1). The Prokaryotic Code is unusual amongst these codes in that its nomenclature was effectively rebooted in 1980, whereby all bacterial names proposed to that point were made null and void due to the high number of synonyms and inadequate or non-uniform descriptions, and an ‘Approved Lists of Bacterial Names’ was established. Names not on those lists lost their standing in nomenclature [78]. All codes have in common the use of type specimens or strains, which serve as a permanent reference for a given species name. However, what constitutes type material (Box 3), the specific ranks used, and rules governing how names are established for each rank vary markedly between the different codes [76, 79, 80]. For example, the Prokaryotic Code requires all names be treated as Latin regardless of their origin and that ranks above genus be based on the stem of the type genus name [76]. By contrast the Virus Code only requires that names be alphabetical, and most recently proposed that higher ranks cannot be based on lower rank names [81, 82].

The complexity of multiple nomenclatural codes and sometimes conflicting application of rules even within one code led to proposals for unification and simplification of the different codes. A leading contender was the Biocode, which proposed to harmonise all biological nomenclature codes under a unified Code largely based on the rules of the Botanical Code [83–85]. However, it was met with a great deal of opposition due to the implicit loss of control by existing nomenclatural committees, and potential confusion created by harmonisation of terms that have different meanings for different codes [86, 87]. A revised draft was published in 2011 but continues to lack consensus support [88]. Another major contender for a unified nomenclature was the PhyloCode proposed in 1998 [89–92], which provided rules for naming clades and species through explicit reference to a phylogeny without the need for a hierarchical taxonomic framework. The plan was to use PhyloCode in parallel with existing Linnaean-based codes, with the goal of replacing them at a later date. In principle, phylogenetic trees provide precise coordinates for taxa, making a classification based on a hierarchical taxonomy redundant [93]. However in practice, uptake of the PhyloCode has not occurred highlighting the reluctance of biologists to move away from the Linnaean system.

Box 2 Cyanobacteria—caught between two Codes Traditionally, Cyanobacteria have been classified as blue-green algae based on their morphological resemblance to algae and photosynthetic pigments, and as a consequence their nomenclature was developed under the Botanical Code as the phylum Cyanophyta [155]. As early as the 19th century, however, microbiologists suggested that Cyanobacteria are more closely related to bacteria than algae [17], which has since been validated by sequence analysis showing that the Cyanobacteria and algae do not even belong to the same domain of life [24, 156, 157]. In 1978, a formal proposal was made to govern the nomenclature of the Cyanobacteria under the provision of the Prokaryotic Code to reflect their natural position as bacteria [158]. This was never formally endorsed by the International Committee on Systematics of Bacteria, and the Cyanobacteria were not included in the 1980 reboot of bacterial nomenclature. Following a possibly unintended modification of the Prokaryotic Code approved in 1999, the Cyanobacteria were included in the Prokaryotic Code, but only a handful of cyanobacterial species names have been validly published under this code [155]. A special committee was established in 2012 to harmonise cyanobacterial nomenclature with the intention to prepare an ‘Approved List of Names of Cyanobacteria’ that would provide a consensus nomenclature acceptable to both botanists and bacteriologists. However, the activity of this committee has been minimal [155]. Over 40 years have passed since the first proposal to include the Cyanobacteria under the Prokaryotic Code yet they are still primarily governed by the Botanical Code due to the differences between the two Codes. An unfortunate consequence of this checkered history is that cyanobacterial nomenclature is conspicuously at odds with evolutionary relationships, as they have been primarily classified on morphological features resulting in numerous polyphyletic taxa [159, 160]. Further controversy has recently erupted around the proposed inclusion of phylogenetically related non-photosynthetic lineages in the phylum [116, 161]. This classification was actually already flying under the radar for many years in 16S rRNA gene databases [125, 129], but became more visible through comparative genomic analyses [161–163].

Box 3 The changing face of type material Type material serves an essential role in traditional nomenclatural systems by providing physically stored material (or descriptions and illustrations) that serve to anchor names in hierarchical classifications as unambiguous points of reference. Type material gives priority to the earliest name of an entity, which prevents naming redundancy [80]. Dried plant specimens were the earliest examples of physical types, although not explicitly incorporated into nomenclatural codes until 1930 [164]. Different codes have different type material requirements, for example the Botanical Code requires non-living specimens with the exception of algae (including cyanobacteria; Box 2) and fungi, which can be preserved in a metabolically inactive (lyophilised) state [80]. The name of the species, which is attached to a specific specimen, becomes validly published by distribution of printed matter through generally accessible libraries or through online publication [80, 165]. By contrast, the Prokaryotic Code requires living axenic strains in dedicated culture collections most conveniently stored as lyophilised material to be designated as types, although written descriptions and illustrations alone were permissible up until January 2001. Since then, for valid publication of a species name, the type strain culture needs to be deposited in at least two publicly accessible culture collections in different countries from which subcultures must be available, and be published in the International Journal of Systematic and Evolutionary Microbiology either as an original article or by inclusion in a Validation List [103]. These stringent requirements mean that the majority of bacteria and archaea cannot currently be accommodated under the Prokaryotic Code due to the inability to bring them into pure culture despite extensive culture-independent characterisation of many as-yet-uncultured species. For this reason, Whitman proposed that sequence data alone, deposited in public sequence repositories, could serve as type material for microorganisms in lieu of cultivated representatives [105].

### (Lack of) nomenclature for uncultured diversity

**Fig. 2:**
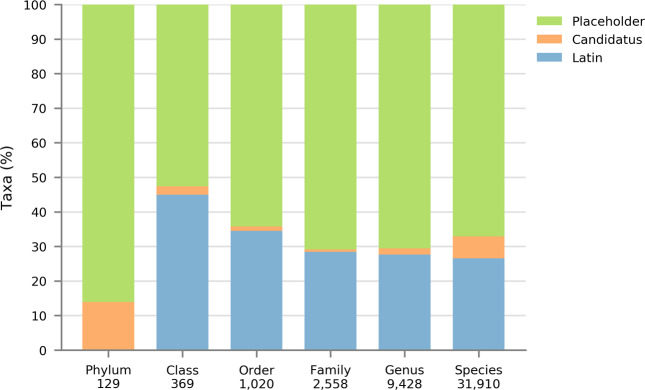
Proportion of Latin, *Candidatus* and placeholder prokaryote names by taxonomic rank based on GTDB Release 05-RS95 [44]. Total number of taxa per rank are shown below each rank name. Most recognised prokaryotic taxa only have placeholder names, and the majority of these fall outside the Prokaryotic Code because they lack cultured representatives (Box 3). Only 7.2% of this excluded fraction have adopted the nomenclatural provisional status of *Candidatus*. The proportion of validly named taxa (Latin names) is likely to fall as MAG sequencing overtakes isolate sequencing. Note that there are no validly published names of phyla as the rank of phylum is not (yet) covered by the rules of the Prokaryotic Code [122].

Detailed molecular characterisation of uncultured microorganisms is a relatively recent innovation due to technological advances (see 16S rRNA and Genome-based classification). Such organisms pose a challenge to the Prokaryotic Code as their names cannot be validly published since species descriptions must be based on pure cultures of type strains (Box 3) and as a consequence they have been outside the rules of the Code [45, 76]. This has resulted in the widespread use of alphanumeric placeholder names for uncultured taxa, which is unregulated and has led to frequent synonymous naming, e.g., Marine Group A/SAR406 [7, 94], GN02/BD1-5 [95, 96] and CD12/BHI80-139 [8]. An early nomenclatural stop-gap for uncultured taxa was proposed in 1994 through the introduction of the provisional status of *Candidatus* [97, 98]. The word *Candidatus* is prefixed to a common name of any rank to indicate the provisional nature of the taxon and has no standing in prokaryotic nomenclature, and therefore no requirement for correct etymology or nomenclature type. Consequently, many *Candidatus* names do not conform to the Prokaryotic Code [99, 100]. Despite these shortcomings, no other proposals have been adopted to accommodate the formalised naming of uncultured taxa, and *Candidatus* has not been widely adopted representing only 4.9% of the 45,414 prokaryotic taxa in the Genome Taxonomy Database (Table 1 and Fig. 2).

*Candidatus* was originally proposed [98] with 16S rRNA environmental surveys in mind. It was expected that their descriptions would be limited in scope compared to isolates, comprising one or at most a few gene sequences, habitat origin (and inferred temperature range) and cell morphology if 16S rRNA-targeted fluorescence in situ hybridisation (FISH) had been successfully applied [36, 97, 101]. However, with the advent of near-complete or even complete MAGs and SAGs [65, 102], and a plethora of techniques able to describe a microorganism’s function without the need for isolation, or even enrichment [103], a *Candidatus* species can be described in great detail. In 2016, it was proposed that gene sequences serve as type material since they are able to provide unambiguous reference points for nomenclature, particularly whole-genome sequences [104, 105]. This would mean that *Candidatus* species (with high-quality genome sequences) could be used as type material and would give them nomenclatural priority (Box 3). Arguments against the use of genome sequences as type material include the lack of deposited physical biomass, lack of uniformly applied genome quality standards, the absence of directly measured phenotypic traits and the potential for nomenclatural chaos due to the much reduced requirements for naming an organism [106, 107]. Given the difficulties in incorporating nomenclature of uncultured microorganisms into the Prokaryotic Code, there have been calls to establish an independent code for these taxa [45, 108]. Proposed minimal standards include genome sequence quality (estimated completeness and contamination), ecological data, a complete 16S rRNA gene sequence, inferred metabolic functions and microscopic identification of the organism using taxon-specific FISH probes or related technique [108]. A key goal of establishing such a parallel code would be that it ultimately converge with the Prokaryotic Code to ensure a unified nomenclature for prokaryotes [45, 108]. A proposal to use sequence data as type material was rejected by the International Committee on Systematics of Prokaryotes (the committee which governs the Prokaryotic Code) in March 2020 [109]. However, if uncultured taxa are ever to be fully integrated into the Prokaryotic Code, sequence data (ideally genome sequences) will have to be accepted as type material, and if this is not possible, a separate nomenclatural code will likely emerge that accepts genomes as type material or does not use type material at all.

### Nomenclatural scaling issues

A recent estimate of the global number of prokaryotic species is 2.2–4.3 million [110], down from previous potentially flawed estimates of trillions [111]. Even with this downwardly revised estimate, there is an enormous gap between millions of species and the current number of species with validly published names (~21K) and genomically described species (~25K) [9, 112]. We are likely to bridge this gap over the coming decades in terms of genome representation, but validation of names of such a large volume of new species via the Prokaryotic Code is not currently possible for uncultured taxa and is time-consuming for microbial isolates (Box 3). This is already being reflected in the high proportion of prokaryotic taxa with placeholder names (Fig. 2). However, it can be reasonably argued that not all identified species need to be given Latin names provided that a systematic taxonomic framework with unique and permanent object identifiers for genomically circumscribed species is established and maintained [1, 44]. Only species that are of sufficient interest to the scientific community would be the subject of more in-depth characterisation and naming. Alternatively, Pallen et al. recently demonstrated the high-throughput generation of grammatically correct Latin names is quite feasible using a combinatorial approach, suggesting that millions of taxa could be named [113]. However, adapting the existing Code or proposing a separate nomenclature for taxa that have not or cannot be obtained in pure culture would still be required.

### Bones of contention between prokaryotic nomenclature and microbial ecology

Microbial ecologists have always appreciated the need to name the microorganisms that they study, however, most are not overly familiar with the rules of nomenclature. This has resulted in a number of points of contention between the two disciplines, which could expand once uncultured taxa are more formally taken into consideration under the Prokaryotic Code or under a new code. First, the Code requires strict adherence to correct Latin grammar, and names are routinely checked for etymological correctness by a small group of experts before publication in the International Journal of Systematic and Evolutionary Microbiology (IJSEM) as original articles or in Validation Lists [76]. *Candidatus* names, by contrast, are not held to these exacting standards as evidenced by a recent compilation in IJSEM, where 35% of 1091 compiled *Candidatus* names required grammatical corrections [100]. Second, since the 1975 revision of the Prokaryotic Code, there is a requirement that higher rank names up to class be formed from the stem of a genus name and a standardised suffix (Rules 8 and 9; [76]). There has been a recent proposal to extend this requirement to the rank of phylum using the suffix *-ota*, which necessitates small variations to numerous existing phylum names, such as Planctomycetes to Planctomycetota and Thermotogae to Thermotogota (Table 1 in [114]). Moreover, the requirement to form higher rank names on subordinate genus stems has resulted in proposals to completely change the names of a number of higher taxa, although there is latitude in the Code to retain older names predating this requirement. For example, it was proposed that the Class Epsilonproteobacteria be renamed to Campylobacteria after the genus *Campylobacter* [115]. Such changes can create unrest amongst microbial ecologists who value continuity of names in the literature ahead of strict compliance with the Prokaryotic Code. Despite these potential shortcomings (from the ecological viewpoint), the great majority of validated higher taxon names satisfy the genus stem requirement with a few well-established and high-profile exceptions such as the proteobacterial classes and class Actinobacteria [114]. However, if the rank of phylum and *Candidatus* taxa are formally recognized, the number of discrepancies and associated name changes will increase.

## A crossroads for prokaryotic taxonomy and nomenclature

Prokaryotic taxonomy and nomenclature are at an interesting crossroads. On the positive side, we have never been better placed to develop a taxonomy based on objective evolutionary relationships using the burgeoning resource of sequenced microbial genomes [108, 116]. Microbial taxonomies have evolved over time in response to improved methodologies (Fig. 1), and it has been argued that for this reason, an official taxonomy should be avoided to prevent the possibility of it becoming methodologically outdated [1]. However, genomes are the most fundamental blueprints of life making it unlikely that a widely accepted alternative methodology resulting in a radically different and improved taxonomy will be developed. Although there are bioinformatic scaling challenges associated with developing a comprehensive genome-based taxonomy, the high degree of concordance between independent initiatives using different combinations of marker genes bodes well for a robust evolutionary framework [57, 117] that could form the basis of a stable taxonomy.

While the idea of a polyphasic approach to taxonomy is understandable, particularly the goal of using multiple features to define ecologically coherent units [118], we believe that genome sequences alone, specifically the subset of conserved vertically inherited core operating genes, should form the basis of a taxonomic framework. All other phenotypic, genotypic and ecological data can then be usefully overlaid onto this framework in order to understand their individual distributions and evolutionary trajectories relative to the species tree. The benefits of a single consistent taxonomy universally accepted by the scientific community would be manifold, including improved interoperability and communication. This was the impetus for developing the GTDB [44] (Table 1), which has a heavy emphasis on inclusion (i.e., using as much high-quality sequence data as possible from both cultured and uncultured taxa) and systematisation (e.g., uniform and reproducible approaches for defining species representatives and ranks, and provision of full taxonomic assignments from domain to species [9, 44]).

A standardised taxonomic framework needs a nomenclature that is similarly reproducible and objective and will scale with the task at hand. The official prokaryotic nomenclature was developed before the advent of large-scale genome sequencing and characterisation of uncultured taxa, and consequently does not cover the uncultured microbial majority. This impasse will need to be overcome either by development of a separate nomenclature based on genome sequences as type material, or a significant modification of the rules governing *Candidatus* taxa in the Prokaryotic Code [45, 105, 108]. If development of a separate nomenclature does become necessary, it could provide an opportunity to take the best elements of the Prokaryotic Code and streamline other parts mired in historical legacy that are not user friendly [1, 119], and do not scale well to the challenge of big sequence data. One example would be simplification or automated formation of names derived from Latin or Greek with correct etymology, which otherwise only a handful of practitioners worldwide are capable of ensuring [120].

On the negative side, adoption of a universal standardised taxonomy will inevitably be accompanied by growing pains. Several industries have become invested in particular taxonomies and associated nomenclature, which do not necessarily follow an evolutionary framework. For example the well-known bacterial genus *Shigella* is phylogenetically intertwined with *Escherichia* and should be made a synonym based on an evolutionary taxonomy; however, it is maintained as a separate genus to avoid confusion in clinical practice [121]. Similarly, the genus *Lactobacillus* has a high profile in the probiotic sector with many species being familiar to a general audience including *L. acidophilus* and *L. casei*. From a phylogenetic perspective, however, the genus is too deep and also polyphyletic. A recent genome-based revision of the taxonomy of *Lactobacillus* divided it into 24 distinct genera [117], which was accompanied by an outreach campaign to educate probiotic consumers endorsed by the International Scientific Association for Probiotics and Prebiotics. Development of an additional nomenclature while presenting an opportunity for modernisation does carry with it the potential negative of interoperability challenges with the existing Prokaryotic Code. However, this is not unprecedented as exemplified by the case of Cyanobacteria (Box 2), and therefore should be manageable with an open dialogue between nomenclatural committees. With careful management and adequate resourcing, a genome-based taxonomy and streamlined nomenclature would be welcomed by a new generation of researchers who use modern approaches to study the microbial world.
